# Behavior of the Genetic Markers at Screening during the First Trimester of Pregnancy in Euploid Fetuses

**DOI:** 10.1055/s-0042-1744461

**Published:** 2022-06-06

**Authors:** Ximena Carolina Romero Infante, Montserrat Uriel, Alejandra Gutiérrez, María Fernanda Hernández, Andres Felipe Hernández, Laura Catalina Jiménez, Jeadran Nevardo Malagón-Rojas, Sara Rincón Franco

**Affiliations:** 1 Universidad El Bosque, El Bosque Research Group of Maternal Fetal Medicine and Gynecology, Ecodiagnóstico El Bosque SAS, Los Cobos Medical Center, Bogotá, Colombia; 2 Universidad El Bosque, El Bosque Research Group of Maternal Fetal Medicine and Gynecology, Bogotá, Colombia; 3 Universidad El Bosque, Research Group of Community Medicine and Collective Health, Bogotá, Colombia; 4 Universidad El Bosque, El Bosque Research Group of Maternal Fetal Medicine and Gynecology, Ecodiagnóstico El Bosque SAS, Bogotá, Colombia

**Keywords:** genetic markers, screening, ultrasonography, chromosomal abnormality, pregnancy, marcadores genéticos, triagem, ultrassonografia, anormalidade cromossômica, gravidez

## Abstract

**Objective** This study aims to describe the behavior of chromosomopathy screenings in euploid fetuses.

**Methods** This is a prospective descriptive study with 566 patients at 11 to 14 weeks of gestation. The associations between ultrasound scans and serological variables were studied. For the quantitative variables we used the Spearman test; for the qualitative with quantitative variables the of Mann-Whitney U-test; and for qualitative variables, the X ^2^ test was applied. Significance was set at *p* ≤ 0.05.

**Results** We have found that gestational age has correlation with ductus venosus, nuchal translucency, free fraction of β subunit of human chorionic gonadotropin, pregnancy-associated plasma protein-A and placental growth factor; there is also a correlation between history of miscarriages and nasal bone. Furthermore, we correlated body mass index with nuchal translucency, free fraction of β subunit of human chorionic gonadotropin, and pregnancy-associated plasma protein-A. Maternal age was associated with free fraction of β subunit of human chorionic gonadotropin and pregnancy-associated plasma protein-A.

**Conclusion** Our study demonstrates for the first time the behavior of the biochemical and ultrasonographic markers of chromosomopathy screenings during the first trimester in euploid fetuses in Colombia. Our information is consistent with international reference values. Moreover, we have shown the correlation of different variables with maternal characteristics to determine the variables that could help with development of a screening process during the first trimester with high detection rates.

## Resumo

**Objetivo** Este estudo tem como objetivo descrever o comportamento do rastreamento de cromossomopatias em fetos euploides.

**Métodos** Trata-se de um estudo prospectivo descritivo com 566 pacientes, entre 11 e 14 semanas de gestação. A associação entre a ultrassonografia e as variáveis sorológicas foi estudada. Para as variáveis quantitativas foi utilizado o teste de Spearman; para as qualitativas com variáveis quantitativas foi utilizado o teste U de Mann-Whitney e para as variáveis qualitativas foi aplicado o teste X2. A significância foi fixada em *p* ≤ 0,05.

**Resultados** Constatou-se que a idade gestacional tem correlação com o ducto venoso, translucência nucal, fração livre da subunidade β da gonadotrofina coriônica humana, proteína plasmática A associada à gravidez e fator de crescimento placentário; há também correlação entre a história de abortos e o osso nasal. Além disso, correlacionamos o índice de massa corporal com translucência nucal, fração livre da subunidade β da gonadotrofina coriônica humana e proteína plasmática A associada à gravidez. A idade materna foi relacionada com fração livre da subunidade β da gonadotrofina coriônica humana e proteína plasmática A associada à gravidez.

**Conclusão** Nosso estudo demonstra pela primeira vez o comportamento dos marcadores bioquímicos e ultrassonográficos de triagem de cromossomas durante o primeiro trimestre em fetos euploides na Colômbia. Nossa informação é consistente com a referência de valores internacionais. Além disso, mostram-se as relações das diferentes variáveis com as características maternas para determinar as variáveis capazes de ajudar no desenvolvimento de um processo de rastreamento durante o primeiro trimestre com alta taxa de detecção.

## Introduction

The chromosomopathies are an important cause of perinatal deaths and child disability. It has been described that approximately 7.9 million children had some type of congenital malformation. 1 Additionally, they are responsible for approximately 270.000 deaths of newborns in the first 28 days. 2 Therefore, the importance of early diagnosis is the early detection of any anatomical and functional alterations that can suggest a genetic abnormality, 3 to provide adequate counseling to parents and offer diagnostic methods that could define the course of the pregnancy and the fetus' prognosis.

Diverse methods have been introduced for diagnosing chromosomal abnormalities. Invasive methods such as chorionic villus biopsy and amniotic fluid sample for doing karyotype have been done for ages and are well known for their accuracy. Nevertheless, there is an estimated risk of pregnancy loss associated with these procedures, between 0.5 and 1.5%, during all three trimesters. 4 5 Therefore, other methods, like the cell-free DNA analysis in maternal blood are used with a detection rate (DR) of 99% for trisomy 21, 96% for fetal trisomy 18, and 91% for trisomy 13, which are the most frequent chromosomal abnormalities. 6 The biggest limitation of these methods are their high cost, which prevents their use as a primary assessment tool for the entire population. 7

The combined screening at 11 to 14 weeks of gestation has appeared as a tool for effective early detection of fetal trisomies and is accessible for all patients. 6 8 This screening includes maternal characteristics, fetal nuchal translucency thickness (NT), fetal heart rate, maternal free fraction of β subunit of human chorionic gonadotropin (free β-HCG), and pregnancy-associated plasma protein-A (PAPP-A), with DRs for trisomy 21 (91%), 18 (97%), and 13 (94%). Additionally, the DR can be improved, and the false positive rate can decrease by including other markers, such as as nasal bone, ductus venosus, tricuspid valve, and placental growth factor (PlGF). 3 6 7 8 9 10 With further benefits of early prediction of other complications such as preeclampsia, intrauterine growth restriction (IUGR), and other anatomical malformations. 6 9

In Colombia, congenital malformations affect 2 to 3% of newborns and are the main cause of mortality for infants under one-year-old. 2 Moreover, only 21.5% of chromosomal abnormalities are diagnosed early and opportunely, 2 with the rest being diagnosed postnatally. The Ministry of Health's guidelines only recommend the measurement of the NT at 11 to 13 + 6 weeks, and ultrasonography to detect abnormalities at 18 to 23 + 6 weeks. 11 12 13 Therefore, it is important to implement a better method to identify women at risk and increase the detection rates for the sake of appropriate evaluations and on-time interventions, as described by the assessment of the combined screening at first trimester for chromosomopathies.

This has not been previously studied in Colombia, and it is necessary to have references of the screening in our population. For that reason, the aim of this study is to describe, for the first time, the behavior of chromosomopathy screening in euploid fetuses at Bogotá, Colombia.

## Methods

A prospective descriptive study was performed through the analysis of pregnant women over 14-years-old, in the first trimester, between 11 and 14 weeks of gestation, who underwent prenatal care in Bogotá, Colombia. This study evaluated 566 pregnant women, between 2014 and 2018, at the Ecodiagnóstico El Bosque SAS Diagnostic Unit Centre, Clínica El Bosque and Subred Integrada de Servicios de Salud Sur Occidente ESE – Unidad de Servicios de Salud Occidente de Kennedy. Women at risk of abortion and who had not completed the screening were excluded from the study.

In the first prenatal control, we collected data regarding race, socioeconomical level, mother's age, single or multiple pregnancy, personal and familiar background, mean blood pressure, cardiac heart rate, weight, height, and body mass index (BMI).

As part of the screening for chromosomopathies, the following biochemical markers were measured: PAPP-A, free β-HCG and PlGF; we analyzed the ultrasonographic findings regarding theNT, absence or hypoplasia of the nasal bone, ductus venosus, and tricuspid insufficiency. These ultrasonographic data were obtained and evaluated by a certified professional. The biochemical markers were taken the same day that the ultrasound scan was performed, and they were processed by using DELFIA XPRESS (PerkimElmer Inc., Waltham, MA, USA). Data from the medical records were typed and validated by two of the researchers in a Microsoft Excel 2019 (Microsoft Corp. Redmond, Washington, USA) database (El Bosque University License).

For the analytical study of the data it was used the Statistical Package for Social Sciences (IBM Corp., Armonk, NY, USA) software, version 22.0 (Universidad El Bosque License), in which the ultrasound scan and serological variables were compared with five maternal variables—gestational age, maternal age, family history of malformations, history of abortions, and BMI—to identify the possible association between variables. For quantitative variables, averages and standard deviation were calculated. Frequencies and percentages were determined for qualitative variables.

To establish association or not between quantitative variables, a normality test was applied. For the comparison between the quantitative variables, it was used the test of Spearman, for the qualitative with quantitative variable it was used the test U of Mann Whitney and for the comparison between qualitative and qualitative variables, the test *X* ^2^ was applied. Significance was set at *p* ≤ 0.05.

This project has the approval of the Ethical Committee of the Universidad El Bosque. The ethical principles for human research from the Helsinki Declaration 14 and the Colombian Resolution 8430 of 1993 15 were considered in the development of this study, which is an investigation with minimal risk.

Written informed consent was obtained from the pregnant women who participated in this study, which allowed the group to use patients' information, while their privacy was respected throughout the study.

A selection bias was present because the patients in this study correspond to a specific population that can't represent all the population of Bogotá. However, the bias was minimized by clearly describing our participants' characteristics and establishing that the results of this study are representative only for the population study.

## Results

There were 566 mother patients and 572 fetuses evaluated (there were six twin pregnancies). The mean maternal age was 27 years (standard deviation, SD = 6.43). We found that 96.3% ( *n* = 551) of the women were mixed race (mestizo), while the remaining 3.67% ( *n* = 21) identified as either white or African-American. The medium and low socioeconomic levels grouped 96% ( *n* = 579) of the study participants ( Table 1 ). The most frequent pathological antecedents were obesity, 8.39% ( *n* = 48), followed by hypothyroidism, 6.12% ( *n* = 35) and hypertension, 2.45% ( *n* = 14) ( Table 1 ).

**Table 1:** Maternal characteristics of the patients

Characteristic		%	95% CI
Maternal age (years)		27.44	(26.89–27.94)
Socioeconomic level	Low	47.7% ( *n* = 270)	(43.59–51.82)
	Medium	48.2% ( *n* = 273)	(44.12–52.35)
	High	4.06% ( *n* = 23)	(2.437–5.69)
Origins	Caucasic	1.94% ( *n* = 11)	(0.80–3.08)
	African-american	1.76% ( *n* = 10)	(0.68–2.85)
	Mixed	96.3% ( *n* = 545)	(94.73–97.85)
Family history of malformations		13.42% ( *n* = 76)	(10.62–16.24)
Body mass index		24.6	(24.27–24.92)
Primipaternity		4.24% ( *n* = 24)	(2.58–5.9)
Primigravida		25.27% ( *n* = 143)	(21.69–28.84)
Multigravida		63.96% ( *n* = 362)	(60–67.91)
Multigravida with history of abortions		4.06% ( *n* = 23)	(2.43–5.69)
Smoking		8.30% ( *n* = 47)	(6.03–10.58)
Exposition hazards and x-rays		3.53% ( *n* = 20)	(2.01–5.05)
Obesity		8.39% ( *n* = 48)	(6.18–10.78)
Hypothyroidism		6.12% ( *n* = 35)	(4.2–8.16)
Hypertension		2.45% ( *n* = 14)	(1.19–3.75)
History of preeclampsia		7.34% ( *n* = 42)	(5.26–9.58)
History of IUGR		3.48% ( *n* = 22)	(2.29–5.47)
History of miscarriages		30.38% ( *n* = 172)	(26.6–34.18)

**Abbreviations:** 95% CI, 95% confidence interval; IUGR, intrauterine growth restriction.

Within the population studied, 8.22% ( *n* = 47) of the mothers had direct exposure to tobacco and only 0.87% ( *n* = 5) had previous exposure to x-rays. Regarding the gynecologic and obstetric history, 63.96% ( *n* = 362) of the maternal patients were multigravidas, while 25.27% ( *n* = 143) were primigravidas. Furthermore, 37.8% ( *n* = 214) had history of abortions; 24.2% ( *n* = 137) had a history of one abortion, and 1.94% ( *n* = 77) of two or more abortions. Compared with the biomarkers studied, it was found that PAPP-A had an average value of 3.19 mUl/ml (SD = 18.30). The average free β-HCG value was 36.43 mUl / ml (SD = 27.90). The mean value of PlGF concentration among the 554 participants with this information available was 31.26 pg / mL (SD = 18.30). Regarding ultrasound variables, the mean fetal heart rate of the 572 fetuses studied was 157.7 beats per minute (SD = 10.05). Regarding the cephalocaudal length, the average found was 66.35 mm (SD = 9.33). The NT had a mean of 1.60 mm (SD = 0.56). The ductus venosus presented an average pulsatility index (PI) of 0.98 (SD = 0.46) ( Table 2 ). The nasal bone was present in 99.8% of the fetuses ( *n* = 571) and the absence of this marker was found in only one fetus. Finally, tricuspid insufficiency did not occur in any if the patients of this study population. This is the first study of chromosomopathies developed in the Colombian population; therefore, it is important to determine what are the values obtained for each variable in a healthy population ( Table 2 ).

**Table 2:** Biochemical and biophysical markers' average in the study population

Variables	Average	95% CI
PAPP-A (mUI/ml)	3.19	(3.03–3.36)
Free β-hCG (mUI/ml)	36.43	(34.14–38.72)
PlGF (pg/ml)	31.26	(29.92–32.59)
Ductus venosus pulsatility index	0.99	(0.95–1.03)
Nuchal translucency (mm)	1.60	(1.2–2.7)
Crown-rump length (mm)	66.35	(65.58–67.11)
Fetal heart rate (lpm)	157.79	(156.98–158.60)

**Abbreviations:** 95% CI, 95% confidence interval; β-HGC, β human chorionic gonadotropin; PAPP-A, pregnancy-associated plasma protein-A; PlGF, placental growth factor.

To define the behavior and correlation of the variables, each variable was analyzed in relation with gestational age, background of abortions, family history of malformations, maternal age, and BMI, which were the most relevant characteristic found in these women, that are associated with the development of chromosomopathies ( Table 3 ). Of the ultrasonographic variables ( Table 3 ) in this study, we found that the ductus venosus had a statistical significative correlation with the gestational age ( *p* = 0.0000) and history of miscarriages ( *p* = 0.013). Additionally, we found a relation between the ductus venosus and BMI but it was not significant. It was not found any case of tricuspid insufficiency; therefore, it was not possible to analyze this variable. The NT had a direct significative correlation with the gestational age and BMI. It was not found other correlations. The absence or hypoplasia of the nasal bone was associated with the family history of malformations (X2 = 308,392; *p* = 0.000). In relation to the biochemicalmarkers ( Table 3 ), it was found that free β-HCG had an inversely significative relation with the gestational age,maternal age, and BMI, and direct correlation with the history of malformations.

**Table 3:** Correlation of the screening variables

Variables	Variable to compare	Correlation coefficient	Statistical significance ( *p* -value < 0,05)
Ductus venosus	Gestational age	−0.161	*p* = 0.000
	History of miscarriages	0.104*	*p* = 0.013
	History of malformations	−0.036**	*p* = 0.971
	Maternal age	−0.003*	*p* = 0.940
	Maternal BMI	−0.004*	*p* = 0.932
Nasal bone	Gestational age	−0.852**	*p* = 0.394
	History of miscarriages	−0.644**	*p* = 0.520
	History of malformations	308.392***	*p* = 0.000
	Maternal age	−1.610**	*p* = 0.107
	Maternal BMI	−0.733**	*p* = 0.464
Nuchal translucency	Gestational age	0.243*	*p* = 0.000
	History of miscarriages	0.045*	*p* = 0.287
	History of malformations	−1.320**	*p* = 0.187
	Maternal age	0.054*	*p* = 0.194
	Maternal BMI	0.119*	*p* = 0.005
Free β-HCG	Gestational age	−0.295*	*p* = 0.000
	History of miscarriages	0.002*	*p* = 0.969
	History of malformations	−2.138**	*p* = 0.033
	Maternal age	−0.102*	*p* = 0.014
	Maternal BMI	−0.202*	*p* = 0.000
PAPP-A	Gestational age	0.360*	*p* = 0.000
	History of miscarriages	−0.048*	*p* = 0.250
	History of malformations	−1.005**	*p* = 0.315
	Maternal age	−0.167*	*p* = 0.000
	Maternal BMI	−0.259*	*p* = 0.000
PlGF	Gestational age	0.364*	*p* = 0.000
	History of miscarriages	−0.038*	*p* = 0.369
	History of malformations	−1.040**	*p* = 0.298
	Maternal age	0.013*	*p* = 0.763
	Maternal BMI	−0.042*	*p* = 0.312

**Abbreviations:** BMI, body mass index; β-HGC, β human chorionic gonadotropin; PAPP-A, pregnancy-associated plasma protein-A; PlGF, placental growth factor. **Notes:** significative *p* -value ≤ 0.05; the correlation coefficient was calculated with the Sperman correlation*, Mann-Whitney u-test**, and chi-square test***

The PAPP-A had a significant direct correlation with the gestational age. A significant statistical inverse correlation of PAPP-A with maternal age and BMI was found. The last variable evaluated was the PlGF, which had a direct significative correlation with gestational age. No other correlations were found ( Table 4 ). 16 17 18 19

**Table 4:** Biochemical and biophysical markers value study versus literature

Variables (Values of reference)	Our results (average)	Literature (Euploid fetuses)
Nuchal translucency	1.60 mm	1.2–2.7 mm ^a^
Ductus venosus pulsatility index	0.99	0.6–3.3 ^b^
Free β-HCG	36.43 mUI/ml	28.05–36.88 mUI/ml ^c^
PAPPA-A	3.19 mUI/ml	0.6–3.3 mUI/ml ^c^
PlGF	31.26 pg/ml	1.01–176.1 pg/ml ^d^

**Abbreviations:** β-HGC, β human chorionic gonadotropin; PAPP-A, pregnancy-associated plasma protein-A; PlGF, placental growth factor. **Notes:** ^a^ Nicolaides et al. (2004); 16 ^b^ Matias et al. (1998); 17 ^c^ Spencer et al. (1999); 18 ^d^ Kasdaglis et al. (2010). 19

## Discussion

The identification of pregnancies with a high risk of developing fetuses with chromosomal abnormalities by non-invasive methods is still a big challenge. However, it facilitates early detection and prevents unnecessary invasive tests in low risk pregnancies. 4 20 Therefore, the early screening for chromosomopathies is the proposed tool, with high detection rates which offer a complete biochemical and ultrasonographic evaluation of the pregnancy for determining the high risk on time. 10 21 22 The data collected is evaluated with the Nicolaides software, which allowed us to calculate the individual risk through a logarithmic method. 10 16

This screening has been deeply described in countries like England, but it has not been studied in countries with lower economical resources such as Colombia, where invasive methods are expensive, nearly inaccessible, and rarely included in health insurance plans. Therefore, the repercussion of this study is high, as it describes for the first time the behavior of the different variables (ultrasonographic and biochemical) of the genetic screening in relation with maternal characteristics in a healthy group of pregnant women in Bogotá, Colombia, to determine the importance and correlation of each test.

The average results found for each variable are not far from what the literature describes, as shown in Table 4 . The ductus venosus PI had a media of 0.98, which was described by Matias et al. 17 as 0.6 to 3.3, and by Kalayci et al. 23 as 0.73 to 1.22. The NT had an average value of 1.64 mm, which is between the normal values described by Nicolaides et al. 24 of 1.2 to 2.7 mm. Regarding the biochemical markers, free β-HCG and PAPP-A had values of 36.43 mUI/ml and 3.19 mUI/ml, respectively, which were also between the values described for this two variables, of 28.05 to 36.88 mU/ml, 25 and 0.6 to 3.3 mUI/ml, respectively. 18 Finally, we evaluated the PlGF as being the newest variable included in the screening; it had a medium value of 31.26 pg/ml, which is also within the values described by Kasdaglis et al., 19 of 1.01 to 176.1 pg/ml. This information means that the values found in this sample of pregnant women have similar normal values to the ones reported in other studies for healthy pregnancies. These data describe the distribution of the biophysical and biochemical markers for chromosomopathies in Colombian pregnant women during the first trimester of pregnancy.

Among the ultrasonographic variables, the ductus venosus (DV) showed an inverse relation with the gestational age, which means that the longer the pregnancy, the lower is the ductus venosus PI. Maiz et al. 26 made a study to evaluate the ductus venosus, in which they found that the reverse wave prevalence is inversely associated with the CRL, which is useful for determining the gestational age. It is important to mention that 65% of the fetuses with trisomy 21 had this alteration, 55% of the fetuses with trisomy 13 and 18, and 75% of those with Turner syndrome. 26 Additionally, the DV is a fundamental variable in the first trimester screening for chromosomopathies, as it has been demonstrated that the evaluation of the DV increased the sensitivity of the screening up to 92 to 96% and reduced the rate of false positive up to 2.4 to 2.6%. 7 26 27

The absence or hypoplasia of the nasal bone in this study was significantly associated with family history of malformations, but it was only found in one patient. There were no further associations with the other variables. There are few studies which describe the role of the nasal bone and in the literature it has been described that it doesn't influence over the other variables of the screening. However, when it is used in combination with maternal age, NT, free β-HCG, and PAPP-A, it rises the DR of this screening up to 90%, with a false positive rate of 0.5%. 28 Moreover, regardless of whether it is directly associated with the modification of other variables in the screening or not, its frequency of presentation is higher in the fetuses with trisomy 21 and other chromosomal abnormalities than in the fetuses with normal genetical studies such as karyotype. 29

On the other hand, the NT is proportionally associated with gestational age and BMI. This association has been described in other study populations, studies from the United Kingdom described a statistically significant correlation between the measure of NT, maternal BMI, smoking status, and Afro-Caribbean ethnicity. 30 Additionally, for a long time the increased NT thickness has been associated with risk of having a genetic alteration. Since 1998, studies have concluded that the screening including maternal age and NT could have a DR of trisomy 21 of 90%, and a false positive of 5%. 31 Nowadays, it is considered an important risk factor, and the prevalence of aneuploidies, especially trisomy 21, is higher when NT is increased. 3 However, the measurement of the NT is also associated with the gestational age and fetal CRL, and its important to determine the distribution values for the population to have a fixed cut-off point according to the gestational age. A study that was done in Taiwan demonstrates the positive correlation between NT and CRL. 32

Of the biochemical markers, free β-HCG and PAPP-A are well now for the genetic screening during the first trimester. In this study, we found a direct relation of free β-HCG with gestational age, maternal age, and maternal BMI, while PAPP-A was inversely associated with maternal age and maternal BMI, and directly related to gestational age. A similar result for PAPP-A was obtained in Australia, where it was found an inverse relation between maternal BMI and the values of free β-HCG and PAPP-A. 33 Spencer et al. 18 also found a direct relation between PAPP-A and gestational age. Moreover, the presence of trisomy 21 has been associated with lower levels of PAPP-A and higher levels of free β-HCG. Regarding trisomy 18 and 13, we found lower levels of PAPP-A and free β-HCG. 3 These biochemical markers are included in the first trimester screening and are useful for obtaining a DR of 90%, 97%, and 92% for trisomy 21, 18, and 13, respectively, with a false positive rate of 3.1% if PAPP-A and free β-HCG are adjusted to gestational age, maternal weight, ethnicity, and smoking habits, among others. 6

Finally, chromosomopathy studies began analyzing PlGF along with the other variables; studies from the United Kingdom describe that PlGF has a statistically significant direct relation with the gestational age, and inverse relation with the maternal BMI. 34 Similarly, Sifakis et al. 35 has described that the P1GF values increased with the gestational age. In this study, we also found a statistically significant positive relation between gestational age and PlGF.

## Conclusion

There is no available information about the behavior of the tests used in the screening for chromosomopathies in the Colombian population. Therefore, this study could be useful for understanding the correlations of the different variables with maternal characteristics, to determine the most helpful variables for developing the first trimester screening in this population. Moreover, this article provides information on the distribution of the biochemical and ultrasonographic markers in euploid fetuses, which could be the first approach to reference values in Colombian pregnant women, agreeing with the international values of reference. We suggest that the studies in this domain should continue to develop the best way to asses an early diagnosis of chromosomopathies, with the aim of doing appropriate, timely interventions and defining the course of the pregnancy and fetus's prognosis.
